# Tai Chi on psychological well-being: systematic review and meta-analysis

**DOI:** 10.1186/1472-6882-10-23

**Published:** 2010-05-21

**Authors:** Chenchen Wang, Raveendhara Bannuru, Judith Ramel, Bruce Kupelnick, Tammy Scott, Christopher H Schmid

**Affiliations:** 1 Division of Rheumatology, Tufts Medical Center, Tufts University School of Medicine, Boston, Massachusetts, USA; 2 Institute for Clinical Research and Health Policy Studies, Tufts Medical Center, Tufts University School of Medicine, Boston, Massachusetts, USA

## Abstract

**Background:**

Physical activity and exercise appear to improve psychological health. However, the quantitative effects of Tai Chi on psychological well-being have rarely been examined. We systematically reviewed the effects of Tai Chi on stress, anxiety, depression and mood disturbance in eastern and western populations.

**Methods:**

Eight English and 3 Chinese databases were searched through March 2009. Randomized controlled trials, non-randomized controlled studies and observational studies reporting at least 1 psychological health outcome were examined. Data were extracted and verified by 2 reviewers. The randomized trials in each subcategory of health outcomes were meta-analyzed using a random-effects model. The quality of each study was assessed.

**Results:**

Forty studies totaling 3817 subjects were identified. Approximately 29 psychological measurements were assessed. Twenty-one of 33 randomized and nonrandomized trials reported that 1 hour to 1 year of regular Tai Chi significantly increased psychological well-being including reduction of stress (effect size [ES], 0.66; 95% confidence interval [CI], 0.23 to 1.09), anxiety (ES, 0.66; 95% CI, 0.29 to 1.03), and depression (ES, 0.56; 95% CI, 0.31 to 0.80), and enhanced mood (ES, 0.45; 95% CI, 0.20 to 0.69) in community-dwelling healthy participants and in patients with chronic conditions. Seven observational studies with relatively large sample sizes reinforced the beneficial association between Tai Chi practice and psychological health.

**Conclusions:**

Tai Chi appears to be associated with improvements in psychological well-being including reduced stress, anxiety, depression and mood disturbance, and increased self-esteem. Definitive conclusions were limited due to variation in designs, comparisons, heterogeneous outcomes and inadequate controls. High-quality, well-controlled, longer randomized trials are needed to better inform clinical decisions.

## Background

Mental illness affects 450 million people worldwide with 25% of the population affected in their lifetimes[1]. It is a leading cause of disability for people aged 15-44[2]. A growing list of psychological states including stress, anxiety, depression and mood disturbance have been linked to many chronic disorders such as coronary heart disease, cancer, diabetes and mental disorders as well as to accidents [3,4]. Mental illness poses significant economic burdens to those involved, reduces productivity and increases health care costs[5]. Thus, there is an urgent need for inexpensive and effective strategies to promote psychological well-being and improve general heath status, especially for people with chronic conditions.

Over the past decade, evidence from epidemiological studies and clinical trials has demonstrated a positive association between physical fitness and psychological health. Numerous studies have shown that physical activity and exercise as well as mind-body practice reduce morbidity and mortality for coronary heart disease, hypertension, obesity, diabetes and osteoporosis, and improve the psychological status of the general population [6-10].

Tai Chi, a form of Chinese low impact mind-body exercise, has been practiced for centuries for health and fitness in the East and is currently gaining popularity in the West. Our previous investigations have shown that Tai Chi has potential benefits in treating a variety of chronic conditions [11-13]. Significant improvement has been reported in balance, strength, flexibility, cardiovascular and respiratory function, as well as pain reduction and improved quality of life [11]. Several recent reviews have suggested that Tai Chi appears to improve mood and enhance overall psychological well-being [11,14,15]. However, convincing quantitative evidence to estimate treatment effects has been lacking. No meta-analysis addressing any psychological outcomes with Tai Chi has ever been published. To better inform patients and physicians, we systematically reviewed the quantitative and qualitative relationship between Tai Chi and psychological health outcomes (stress, anxiety, depression, mood and self-esteem) by critically appraising and synthesizing the evidence from all published studies of healthy and chronically ill populations in the East and West.

## Methods

### Data sources and searches

We conducted a comprehensive computerized search of the medical literature using 8 English databases: MEDLINE (from 1950), PsycINFO (from 1806), CAB (from 1910), Health Star (from 1966), Cochrane Database of Systematic Reviews (from 1991), CINAHL (from 1982), Global Health (from 1910) and Alt HealthWatch (from 1969). We also searched 3 major Chinese databases recommended by domain experts in evidence-based medicine in China. These included: China Hospital Knowledge Database (from 1994), China National Knowledge Infrastructure (from 1915) and WanFang Data (from 1980) through March 2009. We also searched reference lists of selected articles and reviews. The search terms for our review included "Tai Chi", "Tai Chi Chuan", "Tai Chi Chih", "ta'i chi," "tai ji," "Tai Ji Quan", and "taijiquan".

### Study selection

Published articles that reported original data of randomized controlled trials (RCT), non-randomized comparison studies (NRS) and observational studies (OBS)[11] were eligible if they clearly defined a Tai Chi intervention [16]. We considered English and Chinese publications with at least 10 human subjects and evaluation of at least 1 of the following psychological health outcomes: (1) ***Psychological stress***--an imbalance between perceived capabilities and situational demands with manifestations in emotional states, as well as physiological, psychological and behavioral responses; (2) ***Anxiety***--an emotional state, characterized by a cognitive component (e.g. worry, self-doubt and apprehension) and a somatic component (e.g. heightened awareness of physiological responses such as heart rate, sweaty palms and tension); (3) ***Depression***--a depressive state diagnosed with standard instruments and/or clinical interviews; (4) ***Mood***--a pervasive and sustained emotion that colors the perception of the world; (5) ***Self-esteem***--a awareness of good processed by an individual and a representation of how positive one feels about oneself in general [17-19]. We excluded articles such as reviews, case reports, and conference proceedings that did not provide primary data.

### Data extraction and quality assessment

We assessed the characteristics of the original research and extracted data based on study design; demographics; type and duration of Tai Chi exercise and controls; the psychological measures of stress, depression, anxiety, mood and self-esteem; results and/or the authors' main conclusions. When data were not provided in publications, we contacted the authors for information. Two reviewers extracted data and assessed trial quality of each study independently. Interrater reliability was satisfactory (r ≥ 90). The methodological quality for the RCTs was evaluated based on the Jadad instrument [20], which takes into account whether a study described randomization, blinding, and withdrawals/dropouts.

### Assessment of effect sizes and statistical analysis

When data were reported, we computed effect sizes (ES) in each study separately for stress, anxiety, depression and mood. ES was determined by calculating the standardized mean difference between groups. Overall outcome was assessed by pooling the ES of each study.

We calculated Hedges' g score for each study as a measure of ES. To correct for small sample size bias we computed the bias-corrected Hedges' g score for each measure. The magnitude of the ES (clinical effects) indicates: 0-0.19 = negligible effect, 0.20-0.49 = small effect, 0.50-0.79 = moderate effect, 0.80(+) = large effect. RCTs used the difference between the treatment and control group means. NRS used within-group difference between pretreatment and post-treatment means. In studies that involved more than one active intervention, we restricted our analyses to the Tai Chi and control groups. In view of significant heterogeneity, random-effect models were used for pooling. Heterogeneity was estimated with the I^2 ^statistic for both RCTs and NRS. All analyses were conducted using Meta-Analyst 3.13 statistical software (Tufts Medical Center, Boston, MA) [21].

## Results

**Figure 1:**
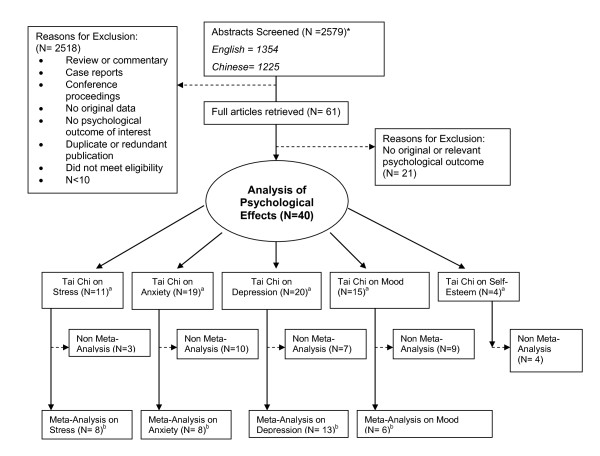
Flow diagram of selection of articles for inclusion* *N = number of studies. ^a^These numbers add up to more than 40 because most studies assessed more than one psychological well-being state. ^b^These numbers add up to more than 21 because most studies assessed more than one psychological well-being state.

We reviewed 2579 English and Chinese articles and retrieved 61 full-text articles for detailed evaluation (Figure 1). Twenty-one studies were eliminated for not reporting original or relevant psychological outcome data. Ultimately, forty studies were identified for data abstraction and critical appraisal. Our search did not identify any unpublished literature.

Table 1 lists the study design and number of studies and participants for each psychological domain. Table 2 describes the 40 studies including 17 RCTs, sixteen NRS and 7 OBS published between 1980 and 2009. They were conducted in 6 countries (USA, China, France, Germany, UK and Australia). There were 3072 healthy individuals (25 studies) and 745 patients with chronic conditions (14 studies); no data were available in 1 study [22]. Mean age ranged from 11 to 92 years, and 62% of participants were female. Various controls were compared among the 17 RCTs, with 6 studies having multiple types of controls. Fourteen of the 16 NRS were self-comparisons while 2 used routine activity as controls. Three of the 7 OBS used routine activity, two used aerobic activity, one was a self-comparison and 1 used the general population as comparisons.

**Table 1:** Summary of reviewed 40 Tai Chi studies (a)

Psychological Profiles	Study Design and Number of Studies and Participants^b^			
	RCT	NRS	OBS	Participants
**Stress**	5	5	1	870
**Anxiety**	5	9	5	1,869
**Depression**	10	6	4	2008
**Mood**	4	8	3	1613
**Self-Esteem**	3	1	0	425

Abbreviations:

N = number of studies. RCT = randomized controlled trial; NRS = nonrandomized comparison study;

OBS = observational study,

^a^*Publication year:*

1980-2009. Language: 8 in Chinese and 32 in English.

Study site: United States (n = 20); China (n = 11); Australia (n = 6); United Kingdom (n = 1); Germany (n = 1); France (n = 1). Most studies assessed more than one psychological well-being state (the numbers add up to more than 40.)

^b^*Study design:*

RCT (N = 17): The investigator manipulates use of the procedure by offering it to one group of people (the exposed group), and offering nothing, a placebo, or some other procedure (s) to another group (the control group) by random allocation. Both groups are then followed to observe the rates of various outcomes.

NRS (N = 16 before and after trials and 2 external comparisons): The investigator causes some subjects to be exposed to the procedure, but does not use random allocation to determine the exposure that includes both an "internal comparison (before and after)" and an "external comparison (unexposed group)."

OBS (N = 7, cross-sectional nature): The investigator does not manipulate use of the procedure, but merely observes and interprets the outcomes.

**Table 2:** Characteristics of 40 articles published in 1980-2009 on Tai Chi and psychological status

Reference^a^	Mean age, yr	Population, n	Duration	Intervention Frequency, style		Psychological Status Measured	Methodological Quality		
				Tai Chi	Control(s)		Randomization^b^	Outcome Assessors Blinded^c^	(% dropout)^d^
**17 Randomized Controlled Trials**									
**Wang et al, 2009, USA**	50	Individuals with FM, 60	12 wks	1 hr, 2×/wk (classical Yang style)	Attention control	Depression (CES-D)	Y	Y	10.6%

**Dechamps et al, 2009, France**	44	Sedentary obese women, 21	10 wks	2 hr, 1×/wk (Yang style)	Exercise Program	Depression (BDI)	Y	Y	0%

**Wang, 2008, USA**	50	Functional class I or II RA, 20	12 wks	1 hr, 2×/wk (classical Yang style)	Attention control	Depression (CES-D)	Y	Y	0%

**Wang et al, 2008, USA**	65	Elderly with symptomatic KOA, 40	12 wks	1 hr, 2×/wk (classical Yang style)	Attention control	Depression (CES-D)	Y	Y	0%

**McCain et al, 2008, USA**	42	Individuals with HIV infection, 252	10 wks	90 min, 1×/wk (focused short form of Tai Chi with 8 movements)	1.Cognitive -behavioral relaxation training 2.Spriritual growth 3.Wait List^e^	Stress (IES, Salivary Cortisol Level)^**f **^, Mood and Emotion (FAHI)	Y	Y	47%

**Fransen et al, 2007, Australia**	70	Elderly with symptomatic hip or KOA,152	12 wks	1 hr, 2×/wk (modified 24 forms Sun style)	1.Hydrotherapy 2.Waiting List^e^	Stress, Anxiety, Depression (DASS 21)	Y	Y	12.5%

**Irwin et al, 2007, USA**	70	Healthy elderly adults, 112	16 wks	40 min, 3×/wk (unspecified style)	Health education	Depression (BDI)	Y	ND	8.9%

**Sattin et al, 2005, USA** **Wolf et al, 2003, USA**	81	Elderly transitioning to frailty, 311	48 wks	60-90 min progression, 2×/wk (6 of the 24 simplified TC forms)	Wellness education	Depression (CES-D)	ND	Y	29.9%

Galantino et al, 2005, USA	(20-60)	Advanced HIV/AIDS, 38	8 wks	1 hr, 2×/wk (unspecified style)	1.Aerobic exercise 2.Usual activity	Anxiety (POMS)	Y	ND	25.5%

**Chou et al, 2004, China**	73	Elderly with depression,14	12 wks	45 min, 3×/wk (18 form of Yang style)	Waiting list	Depression (CES-D-Chinese Version)	ND	Y	ND

Mustian et al, 2004, USA	52	Breast cancer survivors, 21	12 wks	1 hr, 3×/wk (15 short form Yang style)	Psychosocial support therapy	Self-Esteem (RSE)	ND	ND	32.3%

**Tsai et al, 2003, China**	52	Healthy elderly, 76	12 wks	50 min, 3×/wk (108 postures Yang style)	Sedentary life control	Anxiety (STAI)	IA	Y	13.6%

**Li et al, 2001, USA**	73	Healthy elderly, 72	24 wks	1 hr, 2×/wk (classical Yang style)	Waiting list	Stress (SEES) Depression (CES-D) Mood and Emotion (PANAS)	ND	ND	26.5%

Kutner et al, 1997, USA	76	Healthy elderly 130	15 wks	1 hr, 2×/wk (10 modified forms Yang style)	1. education control 2. balance training	Self-Esteem (RSE)	ND	ND	35%

**Sun et al, 1996, USA**	(60-79)	Healthy elderly Hmong immigrants, 20	12 wks	2 hrs, 1×/wk for 10 sessions (unspecified style)	Routine physical activity	Stress (SPS, Body Temperature^f^)	ND	ND	0%

Brown et al, 1995, USA	53	Healthy adults, 135	16 wks	45 min, 3×/wk (unspecified style--"mindful exercise")	1.moderate intensity walk 2.low intensity walk 3.low intensity walk & relaxation 4.usual lifestyle behaviors	Anxiety, Depression, Mood and Emotion (STAI, POMS, Tukey multiple comparison test, LSES, PANAS) Self-Esteem (RSE, SPES and BCS)	ND	ND	25%

Jin, 1992, Australia	36	Healthy adults, 96	1 hr	Single 1 hr session (long form, Yang style or Wu variation of Yang style)	1.TC meditation 2.brisk walking 3.neutral reading	Stress (Salivary Cortisol Level^f^) Anxiety (STAI Y-1) Mood and Emotion (POMS)	ND	Y	ND

**16 Non-Randomized Comparison Studies**									

Lee et al, 2007, Hong Kong, China	83	Healthy elderly, 139	26 wks	1 hr, 3×/wk (unspecified style)	Usual activity	Self-Esteem (SSES-Chinese Version)	N	ND	ND

**Esch et al, 2007, Germany**	28	Healthy young adults, 9	14 wks	90 min, 1×/wk for 12 sessions (Yang style)	Self-comparison	Stress (Perceived Mental Stress (VAS), Salivary Cortisol level) ^**f**^	N	ND	57.1%

**Robins et al, 2006, USA**	42	HIV disease, 59	10 wks	1 hr, 1×/wk (8 movements short form, unspecified style)	Self-comparison	Stress (FAHI, IES)	N	ND	ND

**Taylor-Piliae et al, 2006, USA**	66	Elderly Chinese with CVD risk factors, 38	12 wks	1 hr, 3×/wk (24-posture short form, Yang style)	Self-comparison	Stress (PSS) Anxiety, Depression, Mood and Emotion (POMS)	N	ND	2.6%

**Chen et al, 2005, China**	54	Healthy elderly, 30	24 wks	1 hr, 4×/wk (simplified 24 forms Yang style & 42 forms TC sword)	Self-comparison	Stress, Anxiety (STAI, POMS) Depression, Mood and Emotion (POMS)	N	ND	ND

Wall, 2005, USA	(11-13)	Healthy children, 11	5 wks	1 hr, 1×/wk (traditional segments of Yang style & MBSR)	Self-comparison	Anxiety (Written subjective statements)	N	ND	ND

**Li, 2004, China**	20	Healthy college students, 66	24 wks	1 hr, 3×/wk (unspecified style)	Self-comparison	Anxiety, Depression (SCL-90)	N	ND	ND

**Taggart et al, 2003, USA**	56	Individuals with FM, 21	6 wks	1 hr, 2×/wk (Yang style short form)	Self-comparison	Anxiety, Depression (FIQ)	N	ND	43.2%

**Hernandez-Reif et al, 2001, USA**	15	Adolescents with ADHD, 13	5 wks	30 min, 2×/wk (unspecified style)	Self-comparison	Anxiety, Mood and Emotion (CTRS-R)	N	ND	ND

Mills et al, 2000, UK	48	Adults with MS, 8	8 wks	6 individual sessions (unspecified style or duration)	Self-comparison	Anxiety, Depression (POMS)	N	ND	33.3%

**Ross et al, 1999, USA**	(68-92)	Healthy elderly, 13	8 wks	1 hr, 3×/wk (unspecified style)	Self-comparison	Mood and Emotion (MAACL-R-composite score)	N	ND	23.5%

**Chen & Sun, 1997, USA**	(50-74)	Healthy adults, 28	16 wks 4^th ^mo to 9^th ^mo follow-up	1 hr, 2×/wk, (simplified 24 forms) 1×/mo during follow-up	Routine activity	Anxiety (TMAS and SAI)	N	ND	22.2%

Gibb et al, 1997, Australia	80	Elderly with dementia (56%) or AD patients (44%), 9	7 wks	13 sessions 2×/wk (unspecified style)	Self-comparison	Mood and Emotion (Biographical Self Reflection Program)	N	ND	10%

Fu et al, 1996, Australia	34	Healthy students & teachers, 90	1 yr	30 min, 6×/wk (simplified 24 forms)	Self-comparison	Mood and Emotion (Records from self- report program)	N	ND	ND

Jin, 1989, Australia	(16-75)	Healthy adults with TC experience, 66	1 hr	Single 1 hr session (Long Form Yang style or Wu variation of Yang style)	Self-comparison	Stress (Salivary Cortisol Level^f^) Depression (POMS) Anxiety (STAI) Mood and Emotion (POMS)	N	ND	ND

Mack, 1980, Australia	ND	Afro-American males, ND	24 wks	ND	Self-comparison	Mood and Emotion (no test reported)	N	ND	ND

**7 Observational Studies**									

Chen et al, 2006, China	(20-35)	Healthy students & teachers, 480	0.5-8 yrs	Over 2 hrs/day (24, 28, 58, 88 forms of Chen, Wu, Yang style & TC sword)	Self-comparison	Mood and Emotion Self-design (10 items)	N	Y	ND

Wang & Wang, 2004, China	62	Healthy elderly, 76	>5 yrs	>30 min, >3×/wk (regular TC & TC sword)	1. some activity, <30 min, <3×/wk 2. other activity, >30 min, >3×/wk	Stress (Chinese Psychological Stress Scores)	N	ND	ND

Yang et al, 2004, China	62	Healthy elderly, and middle-aged, 373	>1 yr	Regular TC (unspecified style)	Routine activity	Anxiety (Zung SAS) Depression (Zung SDS)	N	ND	ND

Bond et al, 2002, USA	37	Healthy adults, 249	>6 mos	Regular TC at least 20 min, 3×/wk (unspecified style)	1.sedentary 2.moderate aerobic activity	Anxiety (STAI)	N	ND	0.8%

Chen et al, 2001, Taiwan, China	74	Healthy elderly, 80	≥1 yr	Regular TC at least 2×/wk & 30 min/session (unspecified style)	No exercise control group	Anxiety, Depression, Mood and Emotion (POMS-SF)	N	ND	ND

Liu & Zhang, 2000, China	(18-20)	Healthy individuals, 150	<1 yr >1 yr >2 yrs	Regular TC (unspecified frequency & style)	General population	Anxiety, Depression (Self-Rating Scale-90)	N	ND	ND

Long et al, 2000, China	62	Healthy adults, 239	1-14 yrs	Regular TC (Yang style)	Routine activity	Anxiety, Depression, Mood and Emotion (POMS)	N	ND	ND

Abbreviations: yr = year; n = only evaluated participants included; FM = Fibromyalgia; wk(s) = week(s); hr = hour; CES-D = Center for Epidemiology Studies Depression index; RA = Rheumatoid Arthritis; KOA = Knee Osteoarthritis; DASS 21 = Depression, Anxiety, Stress Scales 21 item questionnaire; min(s) = minute(s); BDI = Beck Depression Inventory; TC = Tai Chi; HIV = Human Immunodeficiency Virus; AIDS = Autoimmune Deficiency Syndrome; POMS = Profile of Mood States; ND = no data; RSE = Rosenberg Self-Esteem scale; STAI = State-Trait Anxiety Inventory; SEES = Subjective Exercise Experience Scale; PANAS = Positive and Negative Affect Schedule; SPS = Self-Perceived Stress score; LSES = Life Satisfaction in the Elderly Scale; SPES = Sonstroem Physical Examination Scale; BCS = Body Cathexis Score; SSES = State Self-Esteem Scale; VAS = Visual Analogue Scale; FAHI = Functional Assessment of HIV Infection; IES = Impact of Events Scale; CVD = Cardiovascular Disease; PSS = Perceived Stress Scale; MBSR = Mindfulness-Based Stress Reduction; SCL-90 = Symptom Checklist-90; FIQ = Fibromyalgia Impact Questionnaire; ADHD = Attention Deficit Hyperactive Disorder; CTRS-R = Conners' Teacher Rating Scale-Revised; MS = Multiple Sclerosis; MAACL-R = Multiple Affect Adjective Checklist-Revised; mo(s) = month(s); TMAS = Taylor Manifest Anxiety Scale; SAI = State Anxiety Inventory; AD = Advanced Dementia; SAS = Self-Rating Anxiety Scale; SDS = Self-Rating Depression Scale.

^a^Studies in **bold **are meta-analyzed

^b^Randomization: Was the study described as randomized (this includes the use of words such as randomly, random, and randomization)? Y = The method to generate the sequence of randomization was described and it was appropriate (table of random numbers, computer generated, etc.); ND = randomization not described; IA = The method to generate the sequence of randomization was described and it was inappropriate (patients were allocated alternately, or according to date of birth, hospital number, etc.); N = non-randomized trial.

^c^Blinding: Was the study described as blind? (Double-blinding is impractical in Tai Chi studies, our modification gave 1 point for proper single blinding of the outcome assessor.) Y = The method of double blinding was described and it was appropriate (identical placebo, active placebo, dummy, etc.); ND = blinding not described.

^d^Dropouts and Withdrawals: Was there a description of withdrawals/dropouts? ND = withdrawals/dropouts not described.

^e^Treatment group compared to Tai Chi in meta-analysis.

^f ^Objective measure.

Nine RCTs reported on randomization; eight of these described an appropriate method, one an inappropriate one. Ten RCTs reported on blinding; in all 10, outcome assessors were blinded. Twelve studies described withdrawals and dropouts. Dropout rates were high with 4 studies reporting dropouts ≥ 25%, and 3 others that reported attrition of 32%, 35%, and 47%. Eleven NRS and 6 OBS reported dropouts, with high rates listed for 3 NRS, reporting 57%, 43% and 33% (Table 2).

**Figure 2:**
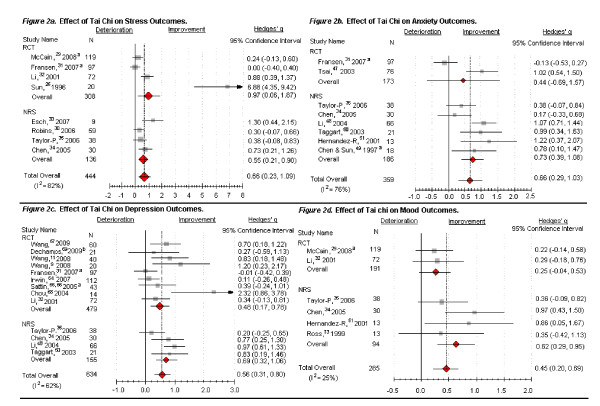
Effects of Tai Chi on stress, anxiety, depression and mood*. RCT = randomized controlled trial; NRS = nonrandomized comparison study (all the meta-analyzed NRS are self-comparison studies). N = number of participants. ^a ^McCain, 2008, included only Tai Chi versus wait list control (n = 119); Fransen 2007, included only Tai Chi versus control group (n = 97); Chen & Sun 1997, included only participants in Tai Chi group as pretreatment, posttreatment (n = 18); Sattin 2005, included only clinically depressed participants in Tai Chi and control arms (n = 43). ^b ^Dechamps, 2009, used an active control compared to Tai Chi. *The magnitude of the effect size (clinical effects) indicates: 0-0.19 = negligible effect, 0.20-0.49 = small effect, 0.50-0.79 = moderate effect, 0.80+ = large effect.

In all, we included 21 trials (12 RCTs and 9 NRS) of 33 that provided data on psychological quantitative measures in the meta-analysis. The 7 OBS were not included in the meta-analysis. Figure 2 displays the overall effects of Tai Chi on stress, anxiety, depression and mood. Table 3 qualitatively assesses the 19 studies that were excluded from the meta-analysis, showing study characteristics, methodology quality, and psychological outcomes. We describe below results for each outcome separately for studies that provided data for meta-analysis and those excluded from meta-analysis.

**Table 3:** Effects of Tai Chi on psychological health (19 non-meta-analyzed studies)

Reference	Control Group(s)	Psychological Status Measured	P-value	Main Conclusions
**5 Randomized Controlled Trials**				

Galantino et al, 2005	1.Aerobic exercise 2.Usual activity	Anxiety (POMS)	0.005	Tai Chi decreased tension-anxiety

Mustian et al, 2004	Psychosocial support therapy	Self-Esteem (RSE)	0.01	Tai Chi improved self-esteem

Kutner et al, 1997	1. Education control 2.Balance training	Self-Esteem (RSE)	NS	Tai Chi improved self-esteem vs. exercise control and vs. education & balance training

Brown et al, 1995	1.Moderate intensity walk 2.Low intensity walk 3.Low intensity walk & relaxation 4.Usual lifestyle behaviors	Anxiety, Depression, Mood and Emotion (STAI, POMS, Tukey multiple comparison test, LSES, PANAS) Self-Esteem (RSE, SPES and BCS)	<0.05	Tai Chi improved anxiety, depression, general mood and mood disturbance and negative affect (women)
			
			NS	Tai Chi improved self-esteem in physical competence (men and women)

Jin, 1992	1.TC meditation 2.Brisk walking 3.Neutral reading	Stress (Salivary Cortisol Level^a^) Anxiety (STAI Y-1) Mood and Emotion (POMS)	<0.005	Tai Chi reduced anxiety vs. neutral reading group
			
			<0.001	All groups reduced stress level and improved total mood disturbance

**7 Non-Randomized Comparison Studies**				

Lee et al, 2007	Usual activity	Self-Esteem (SSES-Chinese Version)	<0.001	Tai Chi improved self-esteem

Wall, 2005	Self-comparison	Anxiety (Written subjective statements)	ND	Students reported feeling calmer, peaceful, relaxed & experienced well-being & improved sleep

Mills et al, 2000	Self-comparison	Anxiety, Depression (POMS)	NS	Tai Chi decreased tension-anxiety
			
			<0.01	Tai Chi decrease depression-dejection

Gibb et al, 1997	Self-comparison	Mood and Emotion (Biographical Self Reflection Program)	ND	Tai Chi improved structured reminiscence with facilitated focused & insightful thinking

Fu et al, 1996	Self-comparison	Mood and Emotion (Records from self- report program)	ND	Tai Chi decreased total mood disturbance

Jin, 1989	Self-comparison	Stress (Salivary Cortisol Level) ^**a **^Depression (POMS) Anxiety (STAI) Mood and Emotion (POMS)	<0.01	Tai Chi decreased stress during and after Tai Chi
			
			<0.001	Tai Chi decreased anxiety and total mood disturbance during and after TC
			
			<0.05	Tai Chi decreased depression during and after Tai Chi

Mack, 1980	Self-comparison	Mood and Emotion (no test reported)	ND	Tai Chi improved mood status

**7 Observational Studies**				

Chen et al, 2006	Self-comparison	Mood and Emotion (Self-design, 10 items)	ND	Tai Chi improved mood status & psychological health

Wang & Wang, 2004	1. >30 min, >3×/wk (regular Tai Chi & Tai Chi sword) 2. some activity, <30 min, <3×/wk 3. other activity, >30 min, >3×/wk	Stress (Chinese Psychological Stress Scores)	<0.01	Tai Chi decreased psychological stress vs. some activity (<30 min, <3×/wk)
			
			NS	Tai Chi decreased psychological stress vs. other activity (>30 min, >3×/wk)

Yang et al, 2004	Routine activity	Anxiety (Zung SAS) Depression (Zung SDS)	<0.01	Tai Chi decreased anxiety and depression scores

Bond et al, 2002	1.Sedentary 2.Moderate aerobic activity	Anxiety (STAI)	<0.05	Tai Chi and moderate aerobic activity decreased state anxiety vs. sedentary group

Chen et al, 2001	No exercise control group	Anxiety, Depression, Mood and Emotion (POMS-SF)	0.000	Tai Chi decreased tension-anxiety, depression-dejection and total mood disturbance

Liu & Zhang, 2000	General population	Anxiety, Depression (Self-Rating Scale-90)	<0.01	Tai Chi decreased anxiety and depression scales

Long et al, 2000	Routine activity	Anxiety, Depression, Mood and Emotion (POMS)	<0.01	Tai Chi decreased anxiety, depression scales and total mood disturbance

Abbreviations: ND = no data; NS = not statistically significant; DASS 21 = Depression, Anxiety, Stress Scales 21 item questionnaire; POMS = Profile of Mood States; RSE = Rosenberg Self-Esteem scale; STAI = State-Trait Anxiety Inventory; LSES = Life Satisfaction in the Elderly Scale; PANAS = Positive and Negative Affect Schedule; SPS = Self-Perceived Stress score; SPES = Sonstroem Physical Examination Scale; BCS = Body Cathexis Score; SSES = State Self-Esteem Scale; FAHI = Functional Assessment of Human Immunodeficiency Virus Infection; SAS = Self-Rating Anxiety Scale; SDS = Self-Rating Depression Scale. ^**a **^Objective measure.

## Tai Chi and Stress

Five RCTs, five NRS and 1 OBS, conducted in 4 countries (USA, Australia, Germany and China) reported the effects of Tai Chi on stress in 870 participants with ages ranging from 16 to 85 years (Table 2). Most studies employed subjective stress measures, including the Depression, Anxiety, Stress Scale [23], Exercise Experiences Scale [24], Self-Perceived Stress score [25], Perceived Mental Stress score, Functional Assessment of HIV Infection [26], Impact of Event Scale [27], Perceived Stress Scale [28], and the Chinese Psychological Stress Scores. Two objective measures collected were body temperature[29] and salivary cortisol levels [30,31].

### Meta-analysis results

Four RCTs and 4 NRS with 444 participants assessed the effects of Tai Chi on stress in individuals with HIV [32,33], elderly with symptomatic hip or knee osteoarthritis [34], healthy participants [29,35-37], and elderly with cardiovascular disease risk factors [38]. Tai Chi was performed between 10 and 24 weeks (60 to 120 minutes, 1 to 4 times per week). We found statistically significant improvements in stress management and psychological distress (ES, 0.66; 95% CI, 0.23 to 1.09) (Figure 2a), with an I^2 ^= 82%. This result remained significant upon dropping the study with the largest effect [29].

### Studies not in meta-analysis

Three studies on stress were not included in the meta-analysis because 1 RCT and 1 NRS treated participants with Tai Chi practice for only one hour [39,40], and the third was an OBS[41] (Table 3). The RCT with 96 healthy adults showed significantly decreased levels of stress in all groups after one hour of intervention (Tai Chi, meditation, brisk walking and neutral reading) [39]. The NRS also reported a single one-hour Tai Chi intervention that significantly reduced stress in healthy adults [40]. The OBS using a Chinese psychological stress questionnaire with 76 healthy Chinese elderly reported that 5 years of regular Tai Chi experience (>30 minutes and >3 times per week) significantly improved stress compared with less physical activity (<30 minutes and <3 times per week). This study, however, found no statistically significant difference between Tai Chi and regular activities of the same duration and frequency [41].

### Summary

Overall, Tai Chi was positively associated with improved in stress levels in healthy adults, patients with HIV-related distress and elderly Chinese with cardiovascular disease risk factors [29,32,33,35-41]. However, the overall study quality was modest with inadequate or no controls in the majority of studies.

## Tai Chi and Anxiety

Five RCTs, 9 NRS and 5 OBS investigated the anxiety-reducing effect of Tai Chi in 1869 people from 4 countries (USA, UK, Australia and China) (Table 2). Seven studies used the Profile of Mood States Anxiety subscale[42] and 6 employed the State-Trait Anxiety Inventory [43]. The remainder used: the Depression, Anxiety, Stress Scale [23], Connors' Teacher Rating Scale-Revised [44], Taylor Manifest Anxiety Scale [45], State Anxiety Inventory[46] and Zung Self-Rating Anxiety Scale [47]. Two disease-specific anxiety measures were used: the Symptom Checklist-90[48] and the Fibromyalgia Impact Questionnaire [49].

### Meta-analysis results

Two RCTs and 6 NRS with 359 participants including patients with symptomatic osteoarthritis [34], healthy adults [37,50-52], elderly with cardiovascular disease risk factors [38], individuals with fibromyalgia [53], and adolescents with ADHD [54] found that Tai Chi practiced 2 to 4 times a week (30 to 60 minutes/time) for 5 to 24 weeks was associated with a significant reduction in anxiety (ES, 0.66; 95% CI, 0.29 to 1.03) (Figure 2b) with I^2 ^= 76%.

### Studies not in meta-analysis

Three RCTs, 2 NRS and 5 OBS were not included in the meta-analysis due to lack of sufficient quantitative data (Table 3). The duration of Tai Chi interventions ranged from 1 hour to 14 years (20 minutes to 1 hour, 1 to 3 times a week). Results from 2 RCTs with 134 subjects reported that Tai Chi practice for a single 1 hour session [39], or twice a week for 60 minutes/time over 8 weeks was associated with a significant reduction in anxiety scores both among HIV patients[55] and healthy adults [39,40,56]. Of note, the third RCT by Brown et al, who randomized 135 healthy adults to a Tai Chi-type activity, moderate or low intensity walking, walking with relaxation, or control reported results by gender [56]. The authors observed a significant decline in anxiety for women, but an insignificant decline in anxiety for men. Wall et al conducted an NRS with 11 6^th ^and 8^th ^graders who performed Yang style Tai Chi and Mindfulness-Based Stress Reduction training (1 hour once a week, for 5 weeks) and found that the participants were "calmer, more peaceful and enjoyed improved sleep relaxation" [57]. The other NRS by Mills et al reported a nonsignificant decrease in tension-anxiety in 8 adults with Multiple Sclerosis [58]. Five large OBS of 1091 healthy adult participants, who practiced Tai Chi between 6 months and 14 years, showed significantly reduced anxiety measures compared to the general population [59], sedentary controls and people engaged in routine or moderate aerobic activity [60-63].

### Summary

Overall, Tai Chi was positively associated with reduced anxiety using one or more anxiety measures, but overall study quality was modest.

## Tai Chi and Depression

Ten RCTs, 6 NRS and 4 OBS examined the effects of Tai Chi on depression in 2008 subjects. Tai Chi intervention ranged from a single 1-hour session to 14 years. Six studies used the Center for Epidemiology Studies Depression Scale [64]. Seven used the Profile of Mood States Depression subscale [42], and the rest tests were the Beck Depression Inventory [65], Fibromyalgia Impact Questionnaire [49], Zung Self-Rating Depression Scale[66] and Self-Rating Scale-90.

### Meta-analysis results

Nine RCTs and 4 NRS in 634 people examined the effects of Tai Chi on depression versus education, routine activity, waiting list and other forms of exercise as well as self-comparison among healthy adults [35,37,51,67-69], individuals with rheumatoid arthritis [12], osteoarthritis [13,34], fibromyalgia [53,70], depression disorders [71], sedentary obese women [72], and elderly Chinese with cardiovascular disease risk factors [38]. Of note, only two studies involved participants with clinically diagnosed depression [68,69,71].

Overall, our analysis suggests that 6 to 48 weeks (40 minutes to 2 hours, 1 to 4 times a week) of Tai Chi practice resulted in significant depression-reduction effects compared to various controls (ES, 0.56; 95% CI, 0.31 to 0.80) (Figure 2c) with I^2 ^= 62%. This result remained significant upon dropping the study with the large effect [71].

### Studies not in meta-analysis

One RCT, 2 NRS and 4 OBS lacked sufficient quantitative detail for analysis (Table 3). Brown et al found a significant decrease in depression for women [56]. The studies by Mills and Jin also reported significant decreases in depression after 8 weeks [58] and an hour of Tai Chi practice, respectively [40]. Four OBS in 842 healthy Chinese subjects showed that regular Tai Chi practice up to 14 years demonstrated statistically significant reductions in depressive symptoms compared with routine activity [59,60,62,63].

### Summary

Overall, evidence from most studies showed that Tai Chi tended to reduce depression. This result was associated with improvement in symptoms and physical function in patients with rheumatoid arthritis and multiple sclerosis, as well as improvement in the immune response of healthy elderly participants. However, the vast majority of the studies suffer from less rigorous designs and were conducted on "healthy" populations with only two studies reporting results on participants diagnosed with clinical depression [68,71].

## Tai Chi and Mood

Evidence on the effects of Tai Chi on mood was examined from 4 RCTs, eight NRS and 3 OBS in 1613 subjects. Duration of Tai Chi practice ranged from 1 hour to 14 years (1 to 7 times a week). The majority of studies reported the total score of the Profile of Mood States scale [42]; other measures were the Functional Assessment of HIV Infection [26], Positive and Negative Affect Schedule [73], Life Satisfaction in the Elderly Scale [74], Symptom Checklist-90 [48], Conners' Teacher Rating Scale-Revised [44], and Multiple Affect Adjective Checklist-Revised [75].

### Meta-analysis results

Two RCTs and 4 NRS assessed the effects of Tai Chi on mood in healthy elderly [35,76], individuals with HIV [32], elderly Chinese with cardiovascular disease risk factors [38], and adolescents with attention-deficit hyperactivity disorder[54]. Tai Chi was performed between 5 and 24 weeks (30 to 90 minutes, 1 to 4 times per week). Tai Chi significantly improved mood compared to various controls with overall ES of (0.45; 95% CI, 0.20 to 0.69), and the I^2 ^= 25% (Figure 2d).

### Studies not in meta-analysis

Two RCTs, four NRS and 3 OBS lacked sufficient quantitative detail for analysis. Brown et al reported that 16 weeks of Tai Chi showed significantly elevated mood for women [56]. Significant improvement in mood was also reported in a RCT following short-term Tai Chi training [39]. Results from 3 NRS reported non-significant improvements in mood following 7 weeks to 1 year of Tai Chi [22,77,78] and one NRS reported a significant decrease in mood disturbance after an hour of Tai Chi practice [40]. Similarly, 3 OBS of 799 subjects reported that 0.5 to 14 years of Tai Chi practice significantly improved mood disturbance in healthy participants [62,63].

### Summary

Overall, evidence suggested short and long-term Tai Chi practice had favorable effects on mood among healthy adults [35,37,39,40,56,62,63,76], elderly with cardiovascular risk factors [38], obese women [32], and adolescents with attention deficit hyperactivity disorder [54]. However, the overall study quality was poor with inadequate or no controls in the majority of studies.

## Tai Chi and Self-esteem

### Meta-analysis results

Only 3 RCTs and 1 NRS evaluated the effects of Tai Chi on self-esteem in 425 subjects and there are no sufficient quantitative data for meta-analysis.

### Studies not in meta-analysis

In these 4 trials, Tai Chi practice lasted from 12 to 26 weeks (45 to 60 minutes, 2 to 3 times per week). Among the measurements employed were: Rosenberg's 10-item Global Self-Esteem Scale [79], the Chinese version of the State Self-Esteem Scale [80], Sonstroem Physical Examination Scale [81] and the Body Cathexis Score [82]. Results from 3 RCTs involving 286 subjects reported that 12 to 16 weeks of Tai Chi was associated with increases in self-esteem scores compared with control groups [56,83,84]. Two RCTs, however, reported nonsignificant results [56,84]. A significant improvement in self-esteem was reported in a recent 26 week NRS that compared Tai Chi to routine activity among 139 Chinese healthy elderly [85].

### Summary

Overall, Tai Chi was positively associated with improvement in self-esteem although no meta-analysis result was provided.

## Discussion

Tai Chi, a form of low impact mind-body exercise, has spread worldwide over the past two decades. This systematic review and meta-analysis summarizes and updates results of the effects of Tai Chi exercise on health outcomes[11] in terms of psychological effects in various populations.

Evidence accrued from clinical trials and observational studies indicates that Tai Chi-- both short and long-term--appears to have mental health benefits in promoting psychological well-being, self-esteem and life satisfaction among healthy subjects and patients with chronic conditions. Specifically, twenty-three of the 33 RCTs and NRS from our quantitative meta-analysis and qualitative evidence synthesis reported that 1 hour to 1 year of regular Tai Chi activity significantly reduced stress, anxiety and depression, and enhanced mood in healthy adults and patients with chronic conditions. The 7 OBS with relatively large sample sizes reinforce the beneficial effects of Tai Chi on psychological health, although bias may be inherent in these observational data.

Our review is congruent with other recent epidemiological reports, experimental trials and literature reviews supporting the fact that physical activity and exercise are associated with better psychological health [8,14,15,86]. Biddle et al recently reviewed evidence on physical activity and exercise in relation to different aspects of mental health. They found that exercise is associated with the strongest anxiety-reduction effects and emphasized the causal link between physical activities and reduction in clinically-defined depression [18]. In particular, evidence from meta-analyses and narrative reviews demonstrates that physical activity and exercise as well as mind-body practice have consistently been associated with positive mood and affect [10,14,15,87-89].

There is insufficient evidence to find any dose-response effect of Tai Chi for psychological outcomes. The studies included in this review exhibit a wide variety of Tai Chi styles, frequency, duration and follow up. The Yang style was used in 17 studies. The majority of studies featured Tai Chi 2 to 3 times per week (frequency), for at least 20 minutes and for an average of 40 to 60 minutes per session (duration). The length of exercise programs ranged from 5 weeks to 1 year, or a single hour for two studies. In the OBS, Tai Chi duration ranged from 6 months to 14 years, and all studies found positive psychological benefits. Few studies reported the intensity and relationship between adherence to Tai Chi and positive psychological effects. Further studies are needed to optimize effective evidence-based dose-response effects and should strictly demand descriptions of intensity, frequency, duration and adherence of the Tai Chi exercise.

Tai Chi appears to be an effective therapeutic modality to improve psychological well-being among various populations. However, it is still difficult to draw firm conclusions. *First*, we did not include any unpublished studies. The overall methodological quality of previous studies is unsatisfactory, consisting mostly of small sized or nonrandomized comparisons. Given the few high quality RCTs available for investigation, our review is limited by wide variations in methodological rigor of clinical trials and observational studies. *Second*, the heterogeneous amalgamation of instruments used to collect clinical psychological health data restricts our ability to evaluate differences in these outcomes. *Third*, it remains unclear whether Tai Chi mind-body exercise provides equal or superior psychological benefits compared to moderate-intensity aerobic exercises. *Fourth*, most studies failed to provide objective measures of stress and anxiety such as salivary cortisol level, blood pressure or heart rate, and some studies only reported a subset of psychological outcomes. Due to the limited physiological variables in our analyses, we were unable to analyze the effect of Tai Chi on physiological effects. *Fifth*, the studies included in the meta-analyses demonstrated a relatively high degree of heterogeneity. Various patient populations were used, and most studies involved healthy people. There were also many variations between the included studies with regard to methodological quality (eg, problems of randomization, allocation concealment, or reporting results), which prohibited us from analyzing the quantitative evidence. However, it is difficult to compare results across studies because they were assessed at different time points. Additionally, all the studies published in mainland China, Hong Kong and Taiwan reported unanimously positive results. Differences in methodological rigor between eastern and western studies may be potential sources of heterogeneity, and publication bias may vary across countries and cultures.

Few published studies have specifically investigated the underlying mechanism of action of Tai Chi's effects on psychological health. Many intermediate, but unidentified, variables may lie along the pathway from Tai Chi to improved psychological well-being. Measures of psychological variables and a multitude of other outcome measures are empirically inter-related, and treatment of each outcome can reciprocally and exponentially improve the other. Improvement of psychological status is also associated with improvement in other clinical outcomes such as arthritic pain as well as health status [90]. The possible mechanisms for enhanced psychological health resulting from Tai Chi mind-body exercise may therefore act through its beneficial influence on biological, physiological, cardiovascular, neurological, and immunological effects as well as overall well-being [11,67,89,91].

## Conclusions

In conclusion, the results of these studies suggest that Tai Chi may be associated with improvements in psychological well-being including reduced stress, anxiety, depression and mood disturbance, and increased self-esteem. High-quality, rigorous, prospective, well-controlled randomized trials with appropriate comparison groups and validated outcome measures are needed to further understand the effects of Tai Chi as an intervention for specific psychological conditions in different populations. Knowledge about the physiological and psychological effects of Tai Chi exercise may lead to new complementary and alternative medical approaches to promote health, treat chronic medical conditions, better inform clinical decisions and further explicate the mechanisms of successful mind-body medicine.

## Competing interests

The authors declare that they have no competing interests.

## Authors' contributions

CW obtained funding for the study. CW, BK and CS designed the study. CW, RB, JR, BK and CS conducted the research. RB and CS conducted the meta-analysis. CW wrote the first draft of the manuscript. CW, CS, RB, JR, BK and TS participated in the revision of subsequent draft. All authors read and approved the final manuscript.

## Pre-publication history

The pre-publication history for this paper can be accessed here:

http://www.biomedcentral.com/1472-6882/10/23/prepub
